# Mass cytometry as a platform for the discovery of cellular biomarkers to guide effective rheumatic disease therapy

**DOI:** 10.1186/s13075-015-0644-z

**Published:** 2015-05-18

**Authors:** Nitya Nair, Henrik E Mei, Shih-Yu Chen, Matthew Hale, Garry P Nolan, Holden T Maecker, Mark Genovese, C Garrison Fathman, Chan C Whiting

**Affiliations:** Department of Microbiology and Immunology, Stanford University, Stanford, CA 94305 USA; Division of Immune Monitoring and Biomarker Development, Aduro BioTech, Inc., Berkeley, CA 94710 USA; Department of Medicine, Stanford University, Stanford, CA 94305 USA; Institute for Immunity, Transplantation and Infection, Stanford University, Stanford, CA 94305 USA

## Abstract

The development of biomarkers for autoimmune diseases has been hampered by a lack of understanding of disease etiopathogenesis and of the mechanisms underlying the induction and maintenance of inflammation, which involves complex activation dynamics of diverse cell types. The heterogeneous nature and suboptimal clinical response to treatment observed in many autoimmune syndromes highlight the need to develop improved strategies to predict patient outcome to therapy and personalize patient care. Mass cytometry, using CyTOF®, is an advanced technology that facilitates multiparametric, phenotypic analysis of immune cells at single-cell resolution. In this review, we outline the capabilities of mass cytometry and illustrate the potential of this technology to enhance the discovery of cellular biomarkers for rheumatoid arthritis, a prototypical autoimmune disease.

## Introduction

### Rheumatoid arthritis pathogenesis and patient response to treatment are heterogeneous

Rheumatoid arthritis (RA) is a chronic, systemic, inflammatory autoimmune disorder that attacks diarthrodial joints leading to cartilage destruction and bone erosion [1]. Similar to other rheumatic diseases, the pathogenesis of RA is multifactorial, multi-staged and characterized by heterogeneous disease manifestations and variations in patient response to therapy [2,3]. The etiopathogenesis of RA is unknown, but numerous factors, such as gene polymorphisms, physiology [4,5], environment, lifestyle [6], the microbiome [7] and gender [8], are implicated in the susceptibility, onset, progress and prognosis of disease. Early diagnosis and treatment improve clinical outcome and may prevent irreversible damage to joints [9]; however, diagnosis tends to occur later in disease and current diagnostics lack sensitivity and specificity [10]. Treatment options for RA patients remain far from optimal as the prescription of ‘biologics’ or small molecules is not guided by molecular diagnosis. Thus, therapies are not tailored to suit the immune status of individual patients. Response rates to treatments range from 60 to 70% and are associated with side effects, while suboptimal treatment regimens and missed opportunities for early treatment may exacerbate symptoms. Most, if not all, autoimmune diseases share a similar degree of heterogeneity in pathogenesis and patient outcome. For many of these diseases, such as systemic lupus erythematosus and primary Sjögren’s syndrome, few approved therapies are currently available.

### Few available biomarkers for rheumatoid arthritis

Several advances have been made in diagnostic and prognostic biomarker research for RA [9], particularly in serological (autoantibody) diagnostics and imaging of inflammation [11]. Serum autoantibodies and cytokines can be used to identify asymptomatic individuals prior to the manifestation of clinical disease [12-14], while predictive markers of severe disease include anti-cyclic citrullinated peptide (CCP), serum rheumatoid factor, elevated levels of acute phase reactants in the presence of cartilage destruction and bone erosion [15]. Autoantibody profiling may guide early intervention; for example, methotrexate treatment of RA patients decreased the incidence of progression from undifferentiated arthritis to clinical RA in anti-CCP-positive individuals [16]. Anti-CCP antibodies have been implicated as a potential biomarker of the response to B-cell depletion therapy in RA patients. miR-146a expression is also upregulated in interleukin (IL)-17-expressing T cells, B cells and macrophages in the synovium and in peripheral blood mononuclear cells of individuals with RA [17]. Cellular biomarkers for rheumatic diseases include activated monocytes in RA [18,19]; however, the sensitivity and specificity of cellular biomarkers in the clinic have yet to be determined. For a comprehensive summary of the status of biomarkers available for RA the reader is referred to several published reviews on this topic [20,21]. The dearth of validated biomarkers for RA and other autoimmune diseases warrants the use of more systematic and comprehensive biomarker discovery approaches.

### Rheumatoid arthritis pathogenesis is mediated by immune cell infiltrates

Disease severity, progression and response to therapy in RA patients are mediated by the activation of inflammatory cells in lymphoid tissues and their infiltration into joints. In RA patients, the synovium is infiltrated with activated T and B lymphocytes, macrophages, mast cells and mononuclear cells that differentiate into multinucleated osteoclasts. This immune infiltrate is accompanied by angiogenesis [22,23], the generation of inflammatory cytokines, including IL-1 and tumor necrosis factor (TNF)-α, an increase in reactive oxygen and nitrogen species in the bone and synovium, activation of chondrocyte catabolic pathways, matrix destruction, and inhibition of new cartilage formation [1,24]. Polymorphonuclear leukocytes in the synovial fluid also contribute to this process [25]. Cytokines such as TNF-α, IL-1 and IL-17 regulate expression of receptor activator of nuclear factor kappa-B ligand, which, when bound to its cognate receptor, receptor activator of nuclear factor kappa-B, on pre-osteoclasts, stimulates osteoclast differentiation and activation. The prolonged activation of osteoclasts can lead to bone destruction in RA patients [26,27]. Moreover, the sustained overproduction of proinflammatory cytokines is a key mechanism contributing to chronic inflammation and progression in RA. This is underscored by the success of neutralizing monoclonal antibodies against these cytokines, or their receptors, such as those that block TNF or IL-6, for effective treatment of RA patients.

RA pathogenesis is associated with T cell activation and proliferation, leading to the secretion of cytokines such as IL-2, interferon-γ, TNF-α and IL-4 [1,28-31], which lead to a stimulation cascade in which other cell types, such as B cells, are activated [32]. B cells are found in the synovium and can differentiate into antibody-secreting plasma cells, and produce a number of cytokines such as IL-10, IL-6 and IL-35 [33]. B cells also interact directly with other cells, such as T cells, and serve as antigen-presenting cells to T cells. B cell aggregates, and their associated cytokines and chemokines, may contribute to the formation of tertiary lymphoid-like structures [34]. The role of B cells in RA pathogenesis is demonstrated in the efficacy of rituximab, which eliminates circulating CD20^+^ B cells but exerts less of an impact on plasmablasts [35] and serum autoantibodies, with some variation according to the specificity [36,37].

Macrophages are key effectors in RA pathogenesis through the production of proinflammatory cytokines such as TNF-α, IL-1, IL-6, IL-8 and granulocyte macrophage colony-stimulating factor (GM-CSF) [38-40] that stimulate cells in the local microenvironment, including fibroblasts and osteoclasts, as well as in distant sites in the body. Macrophages secrete cytokines that stimulate hepatocytes to produce acute phase response proteins, such as C-reactive protein. In addition, macrophages secrete prostaglandins, leukotrienes, nitric oxide, and other pro-inflammatory mediators with local and systemic effects. A decrease in the number of macrophages in the sublining of synovial tissue obtained by needle biopsy may serve as an early biomarker of therapeutic efficacy in RA patients [41]. Synovial fibroblasts secrete inflammatory cytokines such as IL-6, IL-8 and GM-CSF, and produce proteases and collagenases [30,42]. Activated neutrophils in the synovial fluid promote joint damage by releasing oxygen-derived free radicals that depolymerize hyaluronic acid and inactivate endogenous inhibitors of proteases [43,44].

In summary, distinct lymphoid and myeloid immune cell types and their functions contribute to RA pathogenesis. Technologies that probe the phenotypic and functional status of a broad range of cell types may improve cellular biomarker discovery for RA.

### The CyTOF platform

Mass cytometry, using the CyTOF® platform (Fluidigm, South San Francisco, CA, USA), relies on the use of antibodies tagged with stable metal isotopes that are used to stain cells, which are in turn analyzed by a time of flight (TOF) mass spectrometer [45,46]. The mass detection range of CyTOF® covers close to 100 mass detection channels (CyTOF® instrument release 1), and offers an increase in the number of measurable parameters per cell, while obviating the need to perform compensation across channels. Since most stable metal isotopes are absent or present in low abundance in biological samples, the background signal associated with this approach is minimal.

In a typical CyTOF® experiment, panels of specific metal-tagged antibodies measuring both surface and intracellular markers are used to stain cells in a workflow similar to that of fluorescence-based flow cytometry (detailed protocol available at [47]). Cell viability may be assessed using rhodium- or iridium-conjugated DNA intercalators, amine-reactive chelators (DOTA-NHS-ester) or cisplatin [48,49]. Cell suspensions are nebulized into single cell-containing droplets, dried in a heated spray chamber and introduced into an inductively coupled argon plasma where they are atomized and ionized. The resulting ion clouds derived from a single cell are analyzed by a TOF mass analyzer. The signal intensity read out for each isotope indicates a particular antibody, which in turn can be correlated to levels of analyte molecules associated with an individual cell [48]. Data from the CyTOF® instrument are exported in the FCS file format and can be analyzed with conventional flow cytometry software, such as FlowJo (TreeStar Inc., Ashland, OR, USA), FCS Express (De Novo software, Glendale, CA, USA) or using web-based data analysis tools such as Cytobank [50].

A typical mass cytometry experiment contains up to 40 measured parameters per cell, yielding a high-dimensional and quantitative analysis of complex cellular networks, and may span multiple patient groups, conditions and time points. The organization, analysis and visualization of mass cytometry datasets are therefore both a challenge and an active area of development. Manual gating is used to verify reliable reporting of markers and to analyze bulk cellular subsets. However, the analysis of multiparametric data using biaxial plots and histograms is tedious, subjective and often fails to reveal unexpected cell populations 'hidden' in high-dimensional data (such as cells expressing unusual marker combinations outside of expected norms). A number of algorithms have been developed or applied to the mass cytometry platform to analyze these complex datasets [51-53]. Here we provide brief descriptions of some of these analytic tools.

#### SPADE

SPADE (spanning-tree progression analysis of density normalized events) was one of the first algorithms developed to analysis mass cytometry data [46,54,55]. In SPADE, density-dependent downsampling and hierarchical, agglomerative clustering of cells are performed. Similar cells cluster together and are arranged into a minimum-spanning tree for two-dimensional visualization. Thus, SPADE provides an instant overview of relative marker expression levels across all cell populations captured by the clustering. The user can switch between markers and different samples analyzed. The advantages of SPADE are that it preserves rare cell types, it can be used to explore the expression of various parameters between clusters and it offers the ability to compare clusters across samples. A drawback of SPADE (and other related algorithms) is the lack of reproducibility since data are randomly sampled from the entire dataset.

#### CITRUS

At present, CITRUS (cluster identification, characterization and regression) is perhaps the most important tool to mine data for biomarker discovery initiatives. Similar to SPADE, CITRUS identifies clusters of phenotypically similar cells in an unsupervised manner and generates maps of cell subsets based on hierarchical clustering [56]. Different statistical tools are implemented in CITRUS, which permit the generation of predictive models based on input data and user-defined stratification criteria, such as patient clinical outcome or disease activities. The cell cluster(s), which are used to form the predictive model, can be traced, their phenotype can be determined and cells of a particular cluster can be further analyzed. The advantage of CITRUS is that it provides a predictive model that can be used to analyze or test newly acquired samples.

#### Principal component analysis

Principal component analysis (PCA) is an established statistical tool that has been applied to mass cytometry datasets [57,58]. PCA calculates linear vectors through all measured parameters and identifies parameter combinations that capture the most variance in the data as well as relationships between samples. This approach derives summary variables, called principal components, that capture as much variation as possible in as few terms as possible to facilitate dimensionality reduction and data visualization. Its limitations are in its inability to capture non-linear relationships and to fully separate many distinct cell populations.

#### viSNE and ACCENSE

Two t-distributed stochastic neighbor embedding (tSNE)-based algorithms are available to visualize high-dimensional single-cell data; namely, viSNE and ACCENSE [59,60]. tSNE is a non-linear dimensionality reduction approach to visualize CyTOF data. viSNE and ACCENSE generate two-dimensional maps, similar to a biaxial scatter plot, that reflect the proximity of cells to one another in high-dimensional space.

### Utility of mass cytometry for biomarker research

In combination with data analysis tools and algorithms, mass cytometry is expected to facilitate the discovery of cellular biomarkers. Based on CyTOF® data, immune cell populations can be quantified at single-cell resolution according to their phenotype and can be defined using over 30 parameters. Antibodies that detect the phosphorylated states of proteins allow for the readout of functional parameters after *in vitro* activation or 'treatment' with drugs. Bodenmiller and colleagues [61] provide an example of how a combination of surface markers and phosphoepitope-specific markers, in conjunction with cell barcoding, can be applied to generate more than 18,000 data points from a single blood sample. Another example of the utility of this platform for biomarker identification is illustrated by Bendall and colleagues [46] in a study in which CyTOF® was used to immunophenotype healthy human hematopoiesis and to identify differential signaling in distinct cell populations in response to cytokines and kinase inhibitors. Signaling phenotypes among specific cell populations induced by clinically meaningful physiologic stimuli were analyzed, and signaling readouts were localized to pathway and cellular subsets. This approach yielded a system-wide view of signaling behaviors in response to drug action and can be adapted to virtually any disease.

Some limitations to the mass cytometry platform prevent its wide-scale adoption. These include the cost of equipment and instrument maintenance. Moreover, light scatter-based measures of cell size and granularity (forward and side scatter) used for exclusion of cellular debris, cell aggregates and discriminating lymphocytes from granulocytes in flow cytometry, are not currently available. In addition, metal reporters do not reach the sensitivity achieved by phycoerythrin or allophycocyanin conjugates used in conventional flow cytometry [52]. Sample collection speed is slower than in conventional flow cytometry (roughly 500 events per second). Furthermore, roughly two thirds of cells ejected from the mass cytometer nebulizer do not reach the detector as ion clouds [53]. Finally, since the cells introduced into the CyTOF® instrument are atomized and ionized, recovery of cells for downstream functional or transcriptional analysis is currently not possible.

### Mass cytometry analysis of solid tissues

Interactions between cells during normal and pathogenic immune responses largely occur in solid tissues rather than in the blood. However, tissue-based biomarkers are more difficult to establish and to transfer into the clinic as sampling requires significant intervention. Analysis of the cellular composition of lymphoid organs and sites of autoimmune attack will aid in understanding the pathogenesis of human autoimmune diseases. The principle of mass cytometry has been applied to immunohistochemistry and imaging analysis [62,63] to facilitate high-dimensional analysis of tissue specimens. Secondary ion mass spectrometry has been used to image antibodies tagged with isotopically purified elemental metal reporters. This multiplexed ion beam imaging (MIBI) technology is capable of analyzing up to 100 targets simultaneously and can be applied to the analysis of standard formalin-fixed, paraffin-embedded tissue sections. MIBI has been used to image breast tumor tissue [62] and may be applied to solid tissues important in autoimmune pathogenesis, such as the bone marrow, spleen, lymph nodes, chronically inflamed tissues such as the inflamed synovium, central nervous system lesions in multiple sclerosis, glandular tissues in Sjögren’s syndrome, inflammatory lesions in autoimmune vasculitis or skin and kidneys in systemic lupus erythematosus.

### Altered signaling response to exogenous TNF-α stimulation after TNF inhibitor treatment measured in a whole blood assay

Up to 40% of individuals with RA demonstrate an inadequate response to anti-TNF-α therapy [64-66]. An even larger proportion of RA patients lose responses over time due to drug resistance or adverse events. Predictive biomarkers may enable identification of non-responders before TNF-α inhibitor (TNFi) therapy is initiated, thereby lowering costs and preventing unwanted complications associated with a therapy that would ultimately not prove effective.

**Figure 1:**
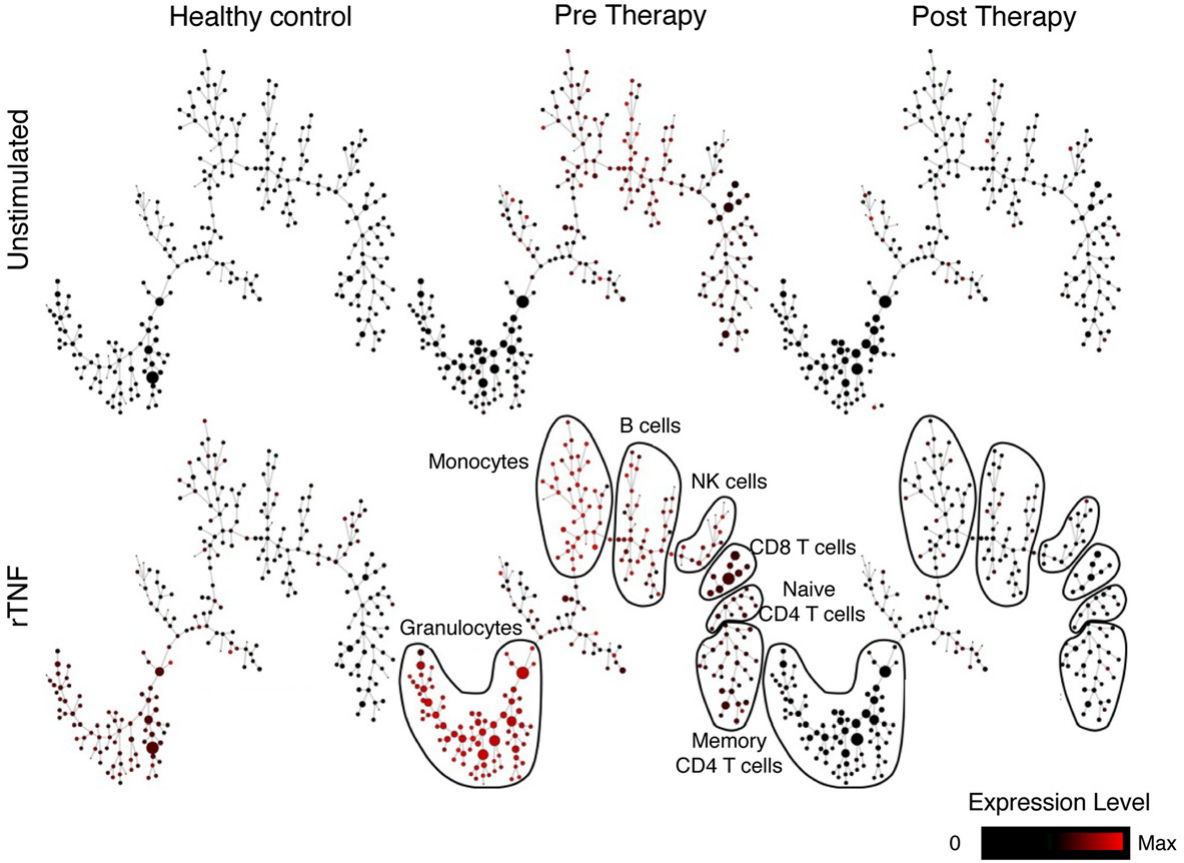
Mass cytometry identification of cell activation and signaling signatures in a rheumatoid arthritis patient treated with tumor necrosis factor-α inhibitor. Whole blood was obtained from a rheumatoid arthritis (RA) patient with a responsive clinical outcome (American College of Rheumatology criteria ACR70) prior to and 1 month following the first application of tumor necrosis factor (TNF)-α inhibitor (TNFi) therapy. A healthy donor was used as a control. Whole blood cells were stimulated *in vitro* with 100 ng/ml TNF-α for 15 minutes at 37°C. Unstimulated cells from the same patient were used as a control. Cells were stained with a panel of 19 metal-tagged antibodies specific to cell surface and intracellular molecules and analyzed by CyTOF. SPADE (spanning-tree progression analysis of density normalized events) was used to cluster cells based on expression of cell surface lineage markers. SPADE analyses shows the level of p38 phosphorylation across annotated cell subsets in unstimulated (top panel) and *in vitro* TNF-α stimulated (bottom panel) cells in healthy donor (left), and RA patient prior to (middle) and 1 month following TNFi treatment (right). Each circular node represents a phenotypically similar population of white blood cells, with the relationship between nodes reflecting the most similar phenotypes to adjacent nodes. The node size represents frequency of that cell population and the node color displays the signal intensity of phosphorylated p38 expression according to the scale. SPADE trees were generated in Cytobank [50]. NK, natural killer; rTNF, recombinant TNF.

In a preliminary experiment, we utilized the CyTOF® platform to analyze the patient immune response to TNF-α prior to and after TNFi treatment. To understand the mechanism of action of TNF blockade (TNFi), we used CyTOF to analyze the key pathways activated in response to TNF signaling and how the activation of these pathways are modulated in response to successful TNFi therapy in different cell subsets in whole blood, prior to and following TNFi treatment. Whole blood was obtained from a healthy donor (untreated) and an RA patient prior to initiation of TNFi treatment. Both subjects were matched in terms of age and sex. The RA patient was receiving steroids and methotrexate at the time of enrollment into the study and was initiated on TNFi therapy (Humira). One month following the first application of therapy, blood was obtained from the patient. The patient’s overall clinical outcome, measured at 3 months after the first TNFi application, was responsive to treatment based on the American College of Rheumatology criteria (ACR70 responder). Peripheral whole blood from the healthy donor and the RA patient (pre- and post-TNFi therapy) was stimulated with recombinant TNF (rTNF; 100 ng/ml) for 15 minutes at 37°C. Unstimulated cells from the same RA patient were used as a control. The cells were stained using a panel of metal-tagged antibodies specific to 19 cell surface markers as well as phosphorylated states of intracellular signaling molecules and then analyzed by CyTOF. SPADE was used to cluster phenotypically similar cells based on the expression of 19 cell surface lineage markers. Major immune cell subsets (granulocytes, monocytes, B cells, natural killer cells, CD8 T cells, naïve CD4 T cells and memory CD4 T cells) were annotated and displayed based on the expression of lineage markers (Figure 1). The expression of phosphorylated p38 was analyzed among clusters within annotated immune cell subsets in unstimulated and in TNF-α-stimulated cells in the healthy donor and in the RA patient prior to and 1 month following the first TNFi application.

A higher basal activation of the TNF receptor (TNFR) pathway(s), reflected by phosphorylation of p38, was observed in the RA patient. Signaling responses to exogenous rTNF were greater in the RA patient than in the normal donor prior to therapy (Figure 1). After a month of TNFi therapy, both the basal activation of TNFR pathways and the response to exogenous rTNF in the patient dropped to levels that were comparable to those observed in the healthy control. In addition, analysis of cell cluster size in the unstimulated samples revealed that the frequency of granulocytes and CD8 T cells was higher in the RA patient prior to TNFi therapy, compared with the healthy donor. One month following the first application of TNFi therapy in the RA patient, the size of cell clusters decreased in the CD8 T-cell compartment but not in the granulocytes in the RA patient. Thus, SPADE was able to reveal quantitative as well as qualitative changes induced by TNFi therapy in this patient.

**Figure 2:**
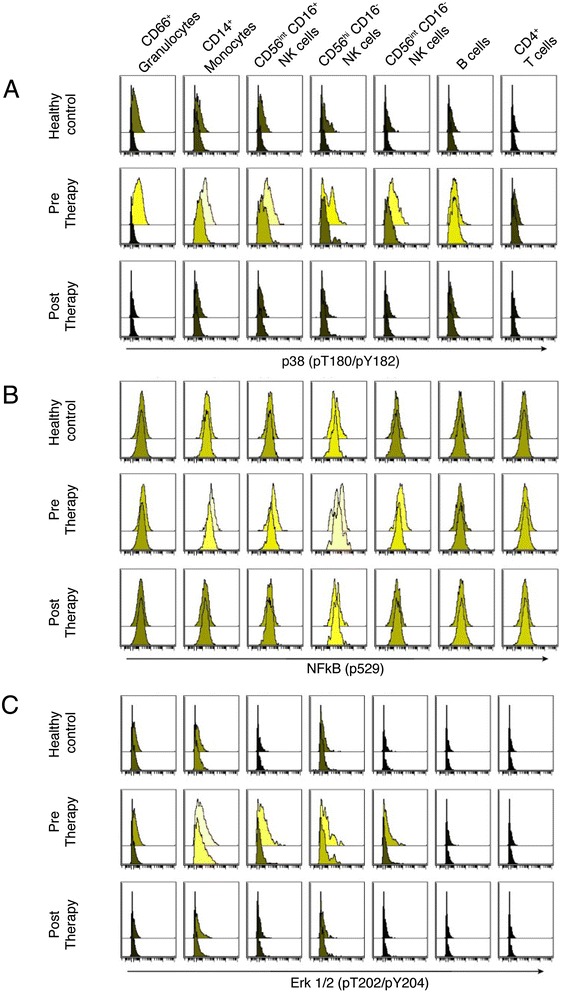
Histogram representation of the levels of phosphorylated p38, NF-kB and Erk1/2. **(A-C)** Levels of phosphorylated p38 **(A)**, NF-kB **(B)** and Erk1/2 **(C)** responding to *in vitro* stimulation with recombinant tumor necrosis factor (TNF)-α in healthy donors (top panel), and rheumatoid arthritis patients prior to (middle panel) and 1 month following TNF-α inhibitor treatment (bottom panel). Lighter colored histograms indicate higher median signal intensity. Within each box, upper histograms represent the stimulated sample; lower histograms represent the unstimulated control sample. All plots were generated in Cytobank [50]. NF, nuclear factor; NK, natural killer.

In addition to phosphorylation of p38, the activation status of the TNFR pathway was also assessed by probing for phosphorylated NF-kB and Erk1/2 levels (Figure 2). Levels of phosphorylated NF-kB were moderately increased by stimulation with rTNF and were more elevated in the patient versus the control sample in some cell subsets (natural killer cells and CD4 T cells) but not in others (Figure 2B), while phosphorylated Erk1/2 levels (Figure 2C) recapitulated changes seen in phosphorylated p38 (Figure 2A). The overall response to TNF-α in the healthy donor was low but evident, characterized by a detectable phosphorylated p38 response particularly in the granulocyte compartment. A smaller response through MAPKAP2 was also detected, whereas Erk showed a low but detectable response to TNF-α in healthy donors.

As expected, our analysis revealed that all three known TNF-induced signaling molecules (p38, NF-kB, Erk1/2) are phosphorylated upon rTNF stimulation in all cell types to varying degrees (Figure 2) in a healthy control and an RA patient. The magnitude to which these signal transducers phosphorylated was enhanced in the RA patient prior to TNFi therapy compared with the healthy control or the patient post-TNFi treatment. The level of activation of all three transducers returned to levels comparable to those observed in the healthy control after 1 month of TNFi therapy. TNF-induced p38 phosphorylation in the granulocyte subpopulations in the whole blood of the RA patient was elevated prior to TNFi treatment, and this level was comparable to that observed in the healthy donor by 1 month post-TNFi therapy (Figure 1).

Several explanations may account for these preliminary observations. The attenuated signal post-TNFi may have been due to *in vitro* neutralization of rTNF by the TNFi drug present in the whole blood. The impact of the cytokine environment in the blood may co-determine the stimulation outcome in RA prior to treatment compared with the control, whereby the decrease in inflammation due to the effect of TNFi treatment reduced the levels of TNF and other inflammatory cytokines that could account for the decreased levels of p38, NF-kB and Erk1/2 phosphorylation after TNFi treatment in the RA patient. Lastly, phosphorylation signals for p38, NF-kB and Erk1/2 may peak similarly but at different time points; this possibility is not accounted for in our preliminary experiment (RA pre-treatment versus control). Work is underway to test these different hypotheses and to extend these initial analyses. These preliminary data illustrate the potential of mass cytometry to identify a previously unappreciated cellular subset, such as granulocytes, that displays functional differences between RA patients compared with healthy donors. In future experiments, this analysis will be extended to additional subjects and staining for TNFR1/2 will be included to decipher which cell type has the greatest response to TNF-α. Our ongoing efforts include the application of CyTOF® to identify cell activation or signaling patterns that may be predictive of clinical outcome in response to TNFi treatment in RA patients.

## Conclusion

Due to the high level of disease heterogeneity in RA and the benefit to be gained from early treatment of patients, the identification of robust biomarkers for diagnosis, prognosis and prediction of successful therapies is paramount. Advances in immune phenotyping technologies, such as mass cytometry, have introduced an unprecedented degree of cell subset resolution that now enables comprehensive profiling of the phenotypic and functional details of patient immune systems. The CyTOF platform is expected to enhance and accelerate cellular and functional biomarker discovery for RA and other autoimmune diseases.

## Note

This article is part of the series ‘*New technologies’*. Other articles in this series can be found at http://arthritis-research.com/series/technology.

## Abbreviations

CCP: cyclic citrullinated peptide
CITRUS: cluster identification, characterization and regression
GM-CSF: granulocyte macrophage colony-stimulating factor
IL: interleukin
MIBI: multiplexed ion beam imaging
PCA: principal component analysis
RA: rheumatoid arthritis
rTNF: recombinant TNF
SPADE: spanning-tree progression analysis of density normalized events
TNF: tumor necrosis factor
TNFi: TNF-α inhibitor
TNFR: TNF receptor
TOF: time of flight
tSNE: t-distributed stochastic neighbor embedding
